# Curcumin Suppresses Crosstalk between Colon Cancer Stem Cells and Stromal Fibroblasts in the Tumor Microenvironment: Potential Role of EMT

**DOI:** 10.1371/journal.pone.0107514

**Published:** 2014-09-19

**Authors:** Constanze Buhrmann, Patricia Kraehe, Cora Lueders, Parviz Shayan, Ajay Goel, Mehdi Shakibaei

**Affiliations:** 1 Institute of Anatomy, Ludwig-Maximilian-University Munich, Munich, Germany; 2 German Heart Institute Berlin, Department of Thoracic and Cardiovascular Surgery, Laboratory for Tissue Engineering, Berlin, Germany; 3 Investigating Institute of Molecular Biological System Transfer, Tehran, Iran; 4 Department of Parasitology, Faculty of Veterinary Medicine, University of Tehran, Tehran, Iran; 5 Gastrointestinal Cancer Research Laboratory, Division of Gastroenterology, Baylor Research Institute and Charles A. Sammons Cancer Center, Baylor University Medical Center, Dallas, Texas, United States of America; Yong Loo Lin School of Medicine, National University of Singapore, Singapore

## Abstract

**Objective:**

Interaction of stromal and tumor cells plays a dynamic role in initiating and enhancing carcinogenesis. In this study, we investigated the crosstalk between colorectal cancer (CRC) cells with stromal fibroblasts and the anti-cancer effects of curcumin and 5-Fluorouracil (5-FU), especially on cancer stem cell (CSC) survival in a 3D-co-culture model that mimics *in vivo* tumor microenvironment.

**Methods:**

Colon carcinoma cells HCT116 and MRC-5 fibroblasts were co-cultured in a monolayer or high density tumor microenvironment model *in vitro* with/without curcumin and/or 5-FU.

**Results:**

Monolayer tumor microenvironment co-cultures supported intensive crosstalk between cancer cells and fibroblasts and enhanced up-regulation of metastatic active adhesion molecules (β1-integrin, ICAM-1), transforming growth factor-β signaling molecules (TGF-β3, p-Smad2), proliferation associated proteins (cyclin D1, Ki-67) and epithelial-to-mesenchymal transition (EMT) factor (vimentin) in HCT116 compared with tumor mono-cultures. High density tumor microenvironment co-cultures synergistically increased tumor-promoting factors (NF-κB, MMP-13), TGF-β3, favored CSC survival (characterized by up-regulation of CD133, CD44, ALDH1) and EMT-factors (increased vimentin and Slug, decreased E-cadherin) in HCT116 compared with high density HCT116 mono-cultures. Interestingly, this synergistic crosstalk was even more pronounced in the presence of 5-FU, but dramatically decreased in the presence of curcumin, inducing biochemical changes to mesenchymal-epithelial transition (MET), thereby sensitizing CSCs to 5-FU treatment.

**Conclusion:**

Enrichment of CSCs, remarkable activation of tumor-promoting factors and EMT in high density co-culture highlights that the crosstalk in the tumor microenvironment plays an essential role in tumor development and progression, and this interaction appears to be mediated at least in part by TGF-β and EMT. Modulation of this synergistic crosstalk by curcumin might be a potential therapy for CRC and suppress metastasis.

## Introduction

Colorectal cancer (CRC) is the third most common cancer in the world and poses major clinical problems due to its high metastasis and recurrence rate [1], [2]. Accumulating evidence suggests that the development and progression of colorectal cancer is due to genetic and epigenetic alterations that are the result of complex interactions of transformed cells with their microenvironment [1], [3]. The tumor microenvironment is regarded as the tumor bed, which comprises of resident components, such as stromal cells and the factors that are stable within the milieu of the stroma, and non-resident components such as different immune cell populations, which influence tumor invasion and metastasis [4]. The synergistic impact of the microenvironment on inflammatory responses and tumor progression is now considered to be an essential feature of carcinogenesis [1], and there is growing interest in the identification of agents that specifically target the pathway interaction between the tumor and stromal cells [5].

It has been proposed that CRC formation arises from a small sub-population of self-renewing tumor stem cells located within the colonic crypt [6], [7]. Indeed, the CRC stem cells (CSC) exhibit properties similar to physiologic stem cells and are responsible for tumor progression [7], [8]. Recently, it has been proposed that CSCs are the unique cell type in the tumor microenvironment that maintain the microenvironment and enhance cancer metastasis and invasion [4], [9]. Further, it has been shown that CSC can directly or indirectly interact with several immune cell populations within the tumor microenvironment, which are thought to markedly influence tumor progression [4].

Identifying agents that are able to suppress the crosstalk between cancer and stromal cells in the tumor microenvironment might be an important therapeutic target for repressing the metastatic potential of CSCs. In order to develop new treatment strategies for CRC, it is therefore essential to study in more detail the interaction of CSCs with the *resident* and *non-resident* components in their microenvironment to elucidate the detailed mechanisms by which CRC development and progression is controlled.

As a large proportion of CRCs are related to environmental factors [1], nutraceuticals offer themselves as ideal candidates to modulate the tumor microenvironment and thus support chemotherapy. Indeed, this is important as more than 15% of patients develop resistance to conventional/current chemotherapy with 5-Fluorouracil (5-FU) and more than 50% of patients develop relapse [10]. We and others have previously shown that nutraceuticals, such as curcumin, can directly influence CRC stem cells by heightening their chemosensitivity to chemotherapeutic treatment, thus markedly increasing positive therapeutic outcome [11]–[13]. Derived from the rhizomes of the plant *curcuma longa*, curcumin (diferuloylmethane) is a naturally occurring yellow polyphenol which mediates its effects by modulation of several important molecular targets including transcription factors (NF-κB, AP-1, β-Catenin), enzymes (e.g. Cox-2, MMPs), pro-inflammatory cytokines (e.g. TNF-α, IL-1β, and IL-6), and cell surface adhesion molecules (e.g. Cadherins, Integrins) [14]–[16]. Modulation of matrix metalloproteinase (MMP) expression in the tumor (micro)-environment is important as MMPs are known markers for CRC progression [17]–[19]. Tumor metastasis and invasion is further influenced by tumor cell interaction with epithelial and endothelial cells. Adhesion and signaling molecules, such as integrins play an important role during CRC progression and metastasis [20], [21]. Further, intracellular adhesion molecule-1 (ICAM-1) has been shown to enhance tumor cell proliferation and invasion and has been identified as being responsible for endothelial adhesion of cancer cells, thus strongly influencing metastatic potential [22], [23]. The tumor microenvironment furthermore induces NF-κB activation which is known to regulate several genes involved in tumor initiation, promotion, and metastasis [24], [25], and induce Wnt activity which plays a critical role in the biology of CRC stem cells [26].

Transforming growth factor-β (TGF-β) is a multifunctional polypeptide that plays an essential role in differentiation, proliferation and embryonic development in normal tissues. Tissue cells synthetize and secrete TGF-β into the microenvironment where it binds to specific TGF-β receptors for paracrine and autocrine signalling. This ligand and receptor complex stimulates intracellular signaling cascades that include the canonical Smad2 signaling pathway [27], which form complexes with Smad4 and accumulates and translocates into the nucleus. In the nucleus, activated Smad complexes regulate the transcription of specific genes and ultimately regulate cell cycle and tissue repair [27]. It is known that TGF-β is a tumor suppressor in normal tissue cells and in early stages of tumor progression. In tumor cells the growth inhibitory effect of TGF-β signalling is dysregulated and it switches from tumor suppressor to tumor promoting factor in different organs [27], [28]. Moreover, it has been reported that the stimulation of TGF-β on stromal cells causes secretion of IL-11 and increases the capability of metastasis of CRC cells whereas animals treated with a specific inhibitor of TGF-β receptor 1 are resilient to metastasis formation [29]. Interestingly, it has been reported that secretion of cytokines and growth factors by stromal cells into a tumor microenvironment triggers an epithelial-to-mesenchymal transition (EMT) that supports drug resistance, tumor recurrence, invasion and metastasis of neoplastic cells [30]. Furthermore, E-cadherin expression is down-regulated during EMT and this can be blocked by zinc finger transcriptional suppressors, such as Slug and this process is reversible [31]–[34].

Characterization of molecular mechanisms involved in the tumor promoting role of TGF-β signaling and EMT in a tumor microenvironment between fibroblasts and tumor cells can help to develop therapeutic strategies against tumor development such as CRC. Therefore, the aim of the presented study was to investigate in more detail the interaction of CRC cells with stromal fibroblasts, activation of cancer-promoting inflammation proteins, paracrine mediators and the modulating effects of curcumin and 5-FU, especially on CRC stem cells and EMT in an *in vitro* cancer microenvironment co-culture, which simulates the *in vivo* tumor microenvironment.

## Materials and Methods

### Antibodies

Monoclonal anti-ALDH1 was obtained from Acris Antibodies GmbH (Herold, Germany). Monoclonal anti-CD133 and anti-CD44 were purchased from Abcam PLC (Cambridge, UK). Anti-β-actin, anti-cyclin-D1, anti-ICAM-1, anti-vimentin, anti-E-cadherin, anti-Slug, anti-TGF-β3, anti-TGF-β3R and anti-p-Smad2 were obtained from Santa Cruz Biotechnology (Santa Cruz, CA, USA). Anti-MMP-1, anti-MMP-9 and anti-MMP-13 were purchased from R&D Systems, Inc., (Heidelberg, Germany). Anti-phospho-specific p65 (NF-κB) and anti-phospho-specific p50 (NF-κB) were obtained from Cell Technology (Beverly, MA, USA). Neutralizing pan-TGF-β antibody, normal rabbit IgG and anti-β1-Integrin were purchased from Sigma-Aldrich Chemie (Munich, Germany). Anti-Ki-67 and secondary antibodies used for fluorescence labelling were purchased from Dianova (Hamburg, Germany). Alkaline phosphatase linked sheep anti-mouse and sheep anti-rabbit secondary antibodies for immunoblotting were purchased from Millipore (Schwalbach, Germany).

### Growth media, chemicals and cytokines

Growth medium (Ham's F-12/Dulbecco's modified Eagle's medium (50∶50) containing 10% fetal bovine serum (FBS), 25 µg/ml ascorbic acid, 50 IU/ml streptomycin, 50 IU/ml penicillin, 2.5 µg/ml amphotericin B, essential amino acids and L-glutamine), Trypsin/EDTA (EC 3.4.21.4) were purchased from Biochrom (Berlin, Germany). 5-FU was purchased from Sigma (Munich, Germany). BCM-95 curcumin, with a purity greater than 95% was purchased from Dolcas Biotech LLC (NJ, USA). This commercial source of curcumin contains three major components: Diferuloylmethane (the most abundant and active component of turmeric) (82%) and its derivatives demethoxycurcumin (15%) and bisdemethoxycurcumin (3%), together referred to as curcuminoids [35], [36]. Curcumin was dissolved in dimethylsulfoxide (DMSO) as a stock concentration of 5000 µM and stored at -80°C. Serial dilutions were prepared in culture medium. 100 mM stock of 5-FU (5-Fluorouracil) was prepared in absolute DMSO and stored at −20°C. The concentration of DMSO was less than 1% of drug treatment. For treatment, 5-FU was diluted in DMEM and added to cultures to give the desired final concentration.

### Cell lines and cell culture

Human colon cancer cells (HCT116) and normal human fibroblast cells (MRC-5) were obtained from the European Collection of Cell Cultures (Salisbury, UK). The cells were maintained in tissue culture flasks with growth medium in a humidified incubator at 37°C in an atmosphere of 95% air and 5% CO_2_. The medium was changed three times a week and cells passaged when 70% confluency was reached. For monolayer tumor microenvironment co-cultures, HCT116 and MRC-5 were co-cultured at a ratio of 1∶1 in monolayer culture and the co-cultures were left for up to 3 days. The supplemented DMEM media was changed every 3–4 days and cells harvested at the time points indicated. All co-cultures were seeded at confluency to ensure optimal contact between the cells. For high density tumor microenvironment co-cultures, HCT116 high density cultures were co-cultured with MRC-5 in monolayer. For formation of high density cultures, a 10µl drop of cell suspension containing around 1 million HCT116 cells was placed on a nitrocellulose filter on top of a steelnet bridge, as previously described [37]. In this system the cells aggregate and are nurtured by diffusion. MRC-5 cells are grown in monolayer on the bottom of the petri dish. This model mimics a three dimensional *in vivo* situation and allows the exchange between resident components and the cancer cells in the tumor microenvironment on the air medium interphase. High density tumor microenvironment co-cultures were either left untreated or treated with curcumin alone (5µM) or 5-FU alone (1, 5, and 10µM) or were pretreated for 4 h with curcumin (5µM) followed by treatment with 5-FU (0.1, 1, 2 and 3µM) for the indicated time. In some experiments, to investigate the role of TGF-β during the crosstalk in the tumor microenvironment co-cultures, the cultures were treated with a neutralizing antibody to TGF-β (10, 20, 30 ng/ml) or control IgG (10, 20, 30 ng/ml) for the indicated time.

### Indirect immunofluorescence microscopy analysis of monolayer and high density tumor microenvironment co-cultures

HCT116 and MRC-5 were co-cultured at a ratio of 1∶1 on glass plates in monolayer or in high density tumor microenvironment co-cultures. After fixation with methanol for 10 min, cells were rinsed three times with PBS and overlaid with bovine serum albumin (BSA) for 30 min. Primary antibodies were diluted 1∶50 in PBS/BSA, incubated overnight at 4°C in a humid chamber, washed three times with PBS/BSA followed by incubation with rhodamine-coupled secondary antibodies (diluted 1∶80 in PBS/BSA) for 1 h at ambient temperature and finally washed again three times with aqua dest. Counter staining was performed with DAPI (4′,6-Diamidino-2-phenylindole, Sigma) to visualize cell nuclei. Slides were covered with fluoromount mountant and examined under a fluorescent microscope (Leica, Germany).

### Toluidine blue staining of monolayer and high density tumor microenvironment co-cultures

MRC-5 cells were cultured in monolayer in high density tumor microenvironment co-cultures with HCT116 cells. High density tumor microenvironment co-cultures were either left untreated, treated with curcumin alone (5µM), 5-FU alone (1µM) or were pretreated for 4 hours with curcumin (5µM) followed by treatment with 5-FU (0.1, 1, 2, 3 µM). HCT116 and MRC-5 cultures were evaluated after 10 days. HCT116 high density tumor microenvironment co-cultures were embedded in Tissue-Tek (Sakura Finetek Europe, The Netherlands), cryopreserved at −80°C and cut into 5–7 µm sections using a cryomicrotome (Zeiss, Germany). Monolayer MRC-5 cells were fixed with methanol for 10 min. HCT116 sections and MRC-5 monolayers were stained with toluidine blue and examined under a light microscope (Leica, Germany).

### Uptake of curcumin in monolayer and high density tumor microenvironment co-cultures

The cellular uptake of curcumin in the monolayer- and high density co-cultures, as described above, was evaluated with the fluorescence method. Briefly, for fluorescence examination HCT116 sections and MRC-5 cultures were fixed with paraformaldehyde, covered with fluoromount mountant and examined under a fluorescent microscope (Leica, Germany).

### Western blot analysis

Whole cell lysates of HCT116 high density mono- or co-cultures were prepared and fractioned by SDS-PAGE [12]. Briefly, cells were rinsed in PBS, and the proteins were extracted with lysis buffer (50 mM Tris/HCl (pH 7.2), 150 mM NaCl, 1% (v/v) Triton X-100, 1 mM sodium orthovanadate, 50 mM sodium pyrophosphate, 100 mM sodium fluoride, 0.01% (v/v) aprotinin, pepstatin A (4 µg/ml), leupeptin (10 µg/ml), and 1 mM phenylmethylsulfonyl fluoride (PMSF)) for 30 min on ice. After adjusting the total protein concentration with bicinchoninic acid system (Pierce) using bovine serum albumin as a standard, equal quantities (500 µg protein per lane) of total proteins were separated by SDS-PAGE under reducing conditions. Separated proteins were transferred to nitrocellulose membranes and incubated in blocking buffer (5% (w/v) skimmed milk powder in PBS, 0.1% Tween 20) for 1 h at ambient temperature. Membranes were incubated overnight with the primary antibody diluted in blocking buffer at 4°C on a shaker, washed 3 times with blocking buffer, and then incubated with the secondary antibody conjugated with alkaline phosphatase for 90 min at room temperature. Membranes were washed 3 times in 0.1 M Tris (pH 9.5) containing 0.05 M MgCl_2_ and 0.1 M NaCl. Specific antigen-antibody complexes were detected using nitroblue tetrazolium and 5-bromo-4-chloro-3-indoylphosphate (*p*-toluidine salt; Pierce, Rockford, IL, USA) as substrates for alkaline phosphatase.

### Statistical analysis

Numerical data are expressed as the mean values (+/−SD) for a representative experiment performed in triplicate. The means were compared using Student's *t* test assuming equal variances. Differences were considered to be statistically significant if the *p* value was less than 0.05.

## Results

To understand some of the key biological behaviors of stromal and tumor cells and to simulate an *in vivo* tumor microenvironment, *in vitro* co-culture systems were established. The focus of this study was to investigate the interaction of HCT116 colorectal cancer cells with human fibroblast MRC-5 cells in a high density co-culture microenvironment model with or without curcumin and/or 5-FU on tumor cell proliferation, tumor-promoting factors, invasion, EMT and colorectal CSCs.

### The intensive crosstalk between HCT116 and MRC-5 cells in the monolayer co-culture microenvironment influences expression of molecules implicated in adhesion, invasion and/or proliferation

**Figure 1:**
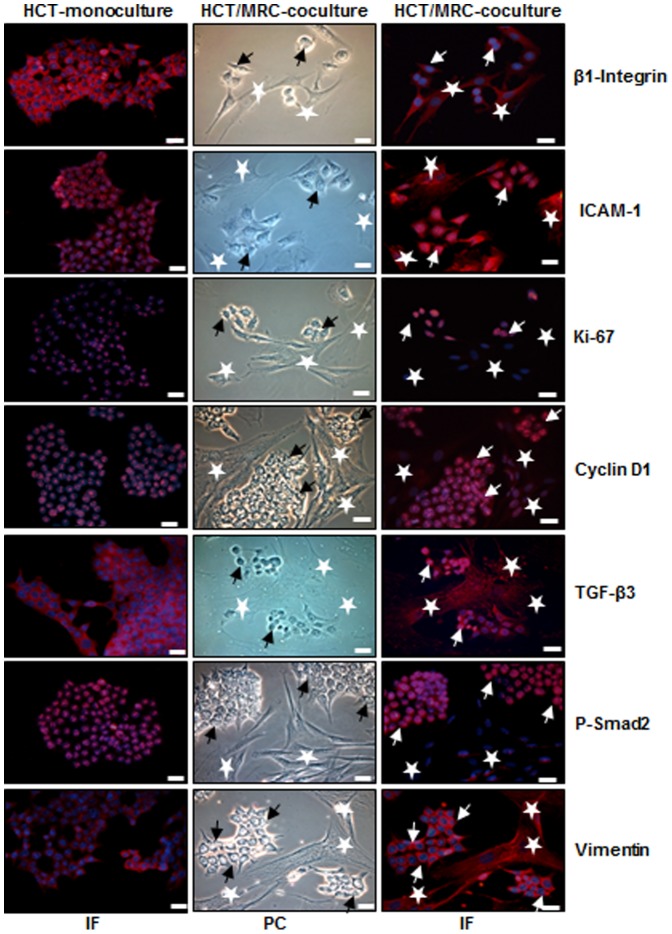
Intensive crosstalk between CRC and MRC-5 cells in the tumor microenvironment. HCT116 cells (arrows) were either cultured alone or were co-cultured with MRC-5 cells (*) at a ratio of 1∶1 for three days on glass plates in monolayer and fixated with methanol. For immunolabeling cells were incubated with primary antibodies against β1-Integrin, ICAM-1, Ki-67, cyclin D1, TGF-β3, p-Smad2 and vimentin) followed by incubation with rhodamine-coupled secondary antibodies and counterstaining with DAPI to visualize cell nuclei. Images shown are representative of three different experiments. PC: phase contrast; IF: immunofluorescence; Magnification 400×; bar = 30 nm.

HCT116 and MRC-5 cells were co-cultured at a ratio of 1∶1 for three days in monolayer and evaluated with light microscopy. HCT116 cells literally accumulated and clustered around the MRC-5 cells searching and establishing close cell-to-cell contact with the MRC-5 cells in the tumor co-culture (Fig. 1, PC). Next, we performed immunofluorescence staining of monolayer co-cultures to investigate whether the observed cellular interaction leads to functional changes in the cells. HCT116 cells in monolayer culture or HCT116 and MRC-5 cells in monolayer co-cultures were labeled with β1-Integrin, ICAM-1, Ki-67, Cyclin D1, TGF-β3, p-Smad2 and vimentin (Fig. 1,IF). We observed strong expression of adhesion and metastatic molecules β1-Integrin, ICAM-1, of active cell cycle proteins Ki-67, Cyclin D1, of TGF-β3, p-Smad2 and of EMT marker vimentin in HCT116 co-cultures compared to HCT116 mono-culture (Fig. 1). Taken together, these findings suggest that stromal cell interaction is crucial in tumor promotion, thereby creating a cellular microenvironment which regulates cancer progression.

### Curcumin and/or 5-FU strongly affect cell integrity in high density tumor microenvironment co-cultures

**Figure 2:**
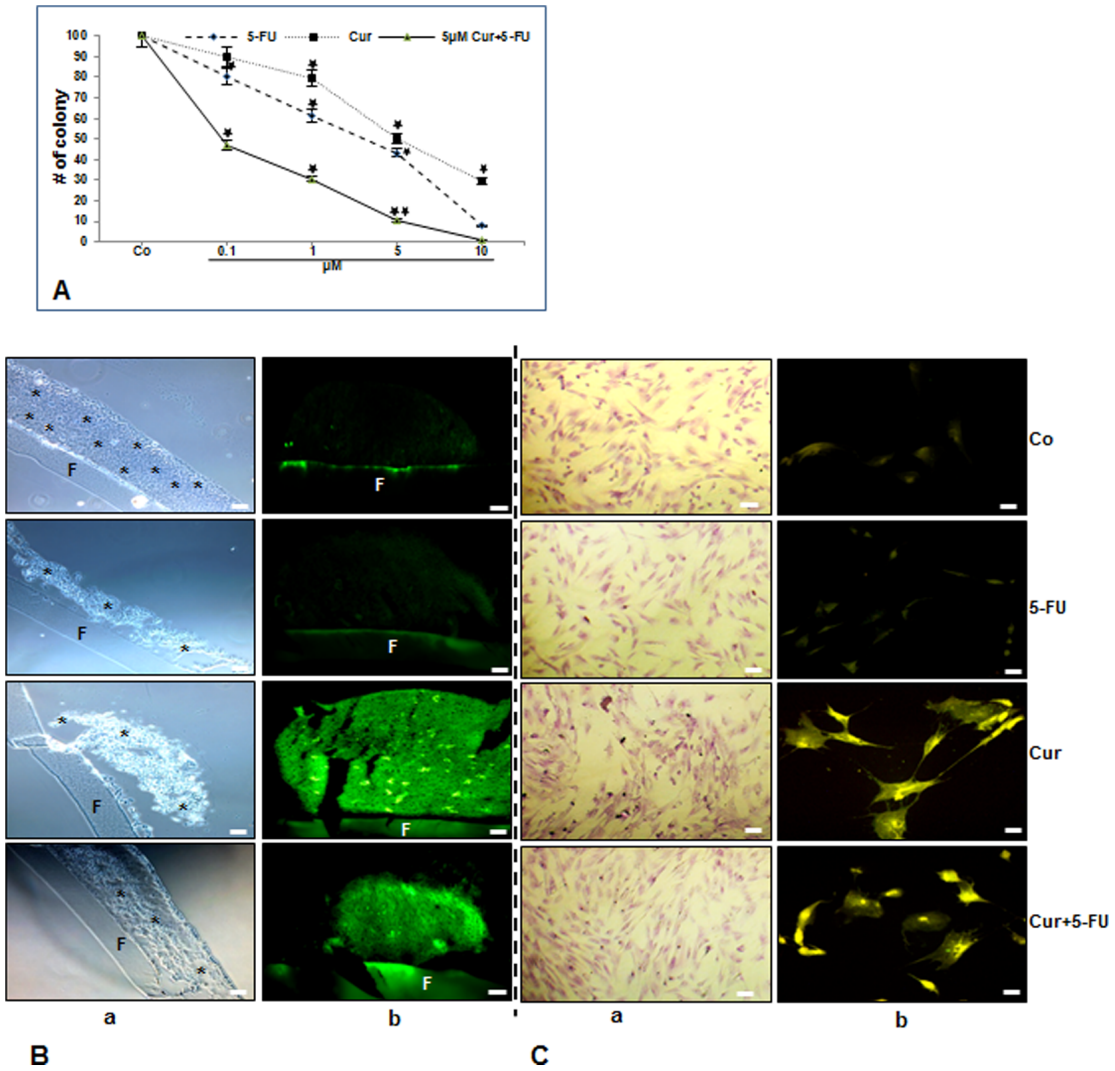
Toxicity of 5-FU, curcumin and the combination treatment on HCT116/MRC-5 cells and cellular uptake of curcumin in these cells in high density and monolayer tumor microenvironment co-culture. A: Quantification of the number of colonosheres was achieved by counting the number of spheroid colonies from 10 microscopic fields in the high density microenvironment co-cultures. Cultures were either left untreated (Co) or were treated with 5-FU (0.1, 1, 5 or 10µM), curcumin (0.1, 1, 5 or 10µM) or were pretreated with curcumin (5µM) for 4 h, and then exposed to 5-FU (0.1, 1, 5 or 10µM) for 10 days and evaluated by light microscopy. Values were compared with the control and statistically significant values with *p<*0.05 were designated by an asterisk (*) and *p<*0.01 were designated by an asterisk (**). Toluidine blue staining profile (2B/C, a) and cellular curcumin uptake (2B/C, b) of HCT116 (B) in high density and in MRC-5 (C) in monolayer co-culture. Tumor microenvironment co-cultures were either left untreated (Co) or were treated with 5-FU (5µM) (5-FU), curcumin (5µM) (Cur) or were pretreated with curcumin (5µM) for 4 h, and then exposed to 5-FU (0.1µM) (Cur+5−FU) for 10 days and evaluated under a light or fluorescent microscope. Images shown are representative of three independent experiments. F =  Filter. (*) =  HCT116 colonosheres. Magnification 4B, 4Ca: 200x, 4Cb: 400x; bar = 30 nm.

Next, we evaluated high density tumor microenvironment co-cultures, HCT116 in high density co-cultured with MRC-5 in monolayer, that mimic the *in vivo* situation to investigate the influences of paracrine therapeutic agents on the crosstalk between the cells and on tumor cell proliferation, tumor-promoting factors, invasion and the formation of tumor spheres, a prominent feature of cancer stem cells. Indeed, it has been reported that normal fibroblast cells are able to induce differentiation of tumor cells such as of a human colon carcinoma cell line [38], [39]. The effects of 5-FU and/or curcumin on cellular integrity and colonosphere formation in tumor microenvironment cultures were evaluated in HCT116 cells after 10 days. The cells were treated with different concentrations of curcumin or 5-FU (0, 0.1, 1, 5 and 10µM) and colonosphere formation was evaluated by light microscopy as described in Materials and Methods. The individual IC_50_ of curcumin or 5-FU were approximately 5µM or 3.5µM, respectively (p<0.05). To evaluate the effect of combined treatment, HCT116 cells were pretreated with 5µM curcumin for 4 h and then co-treated with different concentrations of 5-FU (0, 0.1, 1, 5 and 10µM) for 10 days. Interestingly, pretreatment with curcumin reduced IC_50_ values for 5-FU to 0.1µM in HCT116 (p<0.05) (Fig. 2A). This suggests that curcumin sensitizes HCT116 cells to 5-FU. Further, toluidine blue staining in control cultures showed that cells formed well developed spheroid colonies during the culture period (Fig. 2B:a). Treatment of HCT116 cultures with 5-FU (5µM) or curcumin (5µM) alone or with curcumin and 5-FU (5µM/0.1µM) was shown to be highly effective in inhibiting colonosphere formation and enhancing disintegration of high density tumor spheres compared with the corresponding controls (Fig. 2B:a). This effect was higher in with curcumin or curcumin/5-FU treated co-cultures (Fig. 2B:a). Next, we investigated the cellular uptake of curcumin by HCT116 in tumor microenvironment co-cultures with fluorescent microscopy. The results showed clearly that curcumin was taken up by all HCT116 cells in curcumin treated microenvironment co-cultures (Fig. 2B:b). To investigate, whether the monolayer MRC-5 cells in the microenvironment co-cultures survive the treatment with 5-FU, curcumin or/and 5-FU, we stained the cells with toluidine blue. As shown in Fig. 2C:a, the MRC-5 cells are well stained which is a sign of vitality. Furthermore, curcumin was taken up by all MRC-5 cells in curcumin treated tumor microenvironment co-cultures (Fig. 2C:b).

### Colon CSCs in CRC cell populations are targeted in high density tumor microenvironment co-cultures by 5-FU, curcumin and the combined treatment

**Figure 3:**
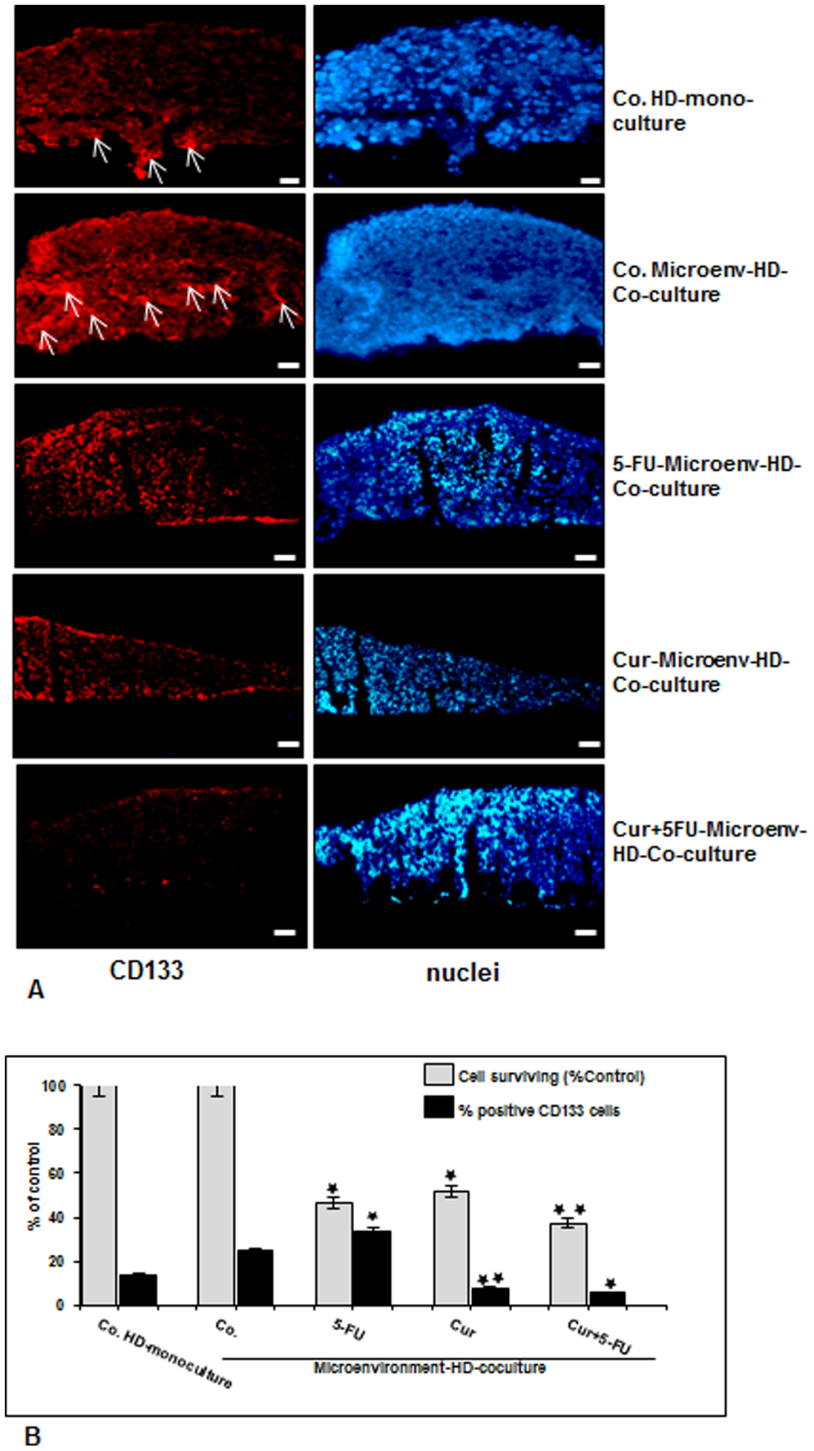
Targeting human colon cancer stem cells (CD133 positive cells) with curcumin and/or 5-FU in high density tumor microenvironment co-culture. A: High density mono-cultures of HCT116 cells were left untreated (A,Co.HD-mono-culture), high density tumor microenvironment co-culture of HCT116/MRC-5 cells were either left untreated (A,Co.Microenv-HD-Co-culture), or treated with 5-FU (5µM) (A, 5-FU-Microenv-HD-Co-culture), curcumin (5µM) (A, Cur-Microenv-HD-Co-culture) or pre-treated with curcumin (5µM) for 4 h, and then exposed to 5-FU (0.1µM) (A, Cur+5FU-Microenv-HD-Co-culture) for 10 days. Immunolabeling was performed with primary antibodies for colon CSC marker (CD133) followed by incubation with rhodamine-coupled secondary antibodies and counterstaining with DAPI to visualize cell nuclei. Images shown are representative of three different experiments. Magnification 400×. bar 30 nm. B: To quantify the amount of CD133-positive cells in high density cultures described above, 100 cells from 15 microscopic fields were counted. The examination was performed in triplicate, and the results are provided as the mean values with S.D. from three independent experiments. Values were compared with the control and statistically significant values with *p<*0.05 were designated by an asterisk (*) and *p<*0.01 were designated by an asterisk (**).

It has been reported that the tumor microenvironment plays an essential role in perpetuation of the CSCs promoting affected by stroma, inflammatory cells, cytokines and growth factors secreted by the stromal fibroblasts [26], [40]. Therefore, we examined the behavior of CSCs within the CRC cell population, high density mono-cultures of HCT116 cells were left untreated. Tumor microenvironment co-cultures of HCT116/MRC-5 cells were either left untreated, or treated with 5-FU (5µM), curcumin (5µM) or pretreated with curcumin (5µM) for 4 h and then exposed to 5-FU (0.1µM) for 10 days. The cultures were subjected to immunofluorescence labeling with primary antibodies for CSC marker (CD133). Slight expression of CD133 was detected in basal control mono-cultures (Fig. 3A). Interestingly, in contrast, CD133 positive cells from the HCT116 cells in microenvironment co-cultures were higher compared to that in control mono-culture (Fig. 3A), indicating the important synergistic role of the crosstalk between HCT116 and MRC-5 cells in supporting tumor promotion. Furthermore, CD133 positive cells from the HCT116 cell line population showed significantly increased survival upon treatment with 5-FU compared with curcumin or/and 5-FU (Fig. 3A). In the presence of curcumin or/and 5-FU, they exhibited marked down-regulation of CD133 positive cells (Fig. 3A and 3B), demonstrating the prominent chemosensitizing effect of curcumin on CSCs. By quantification, we confirmed that the number of CD133-labeled cells increased in the HCT116 microenvironment co-cultures compared to control tumor mono-culture (Fig. 3A), and CD133 positive cells increased in the surviving cell population upon treatment with 5-FU, but not with curcumin or the combined treatment (Fig. 3B).

### Curcumin has potent chemosensitization effect on colon cancer stem cells in CRC cell populations in high density tumor microenvironment co-cultures

**Figure 4:**
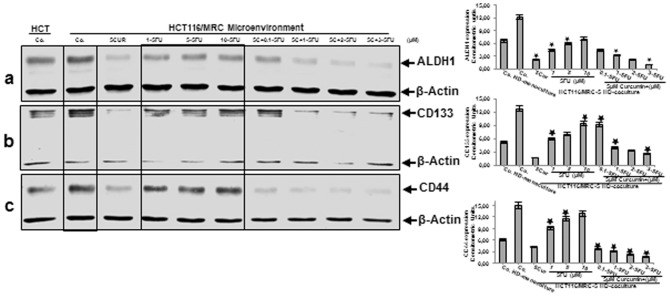
Curcumin sensitizes colon cancer stem cells to 5-FU in the high density tumor microenvironment co-culture. HCT116 high density mono-cultures were either left untreated (HCT, Co.) or were co-cultured with MRC-5 in tumor microenvironment. Tumor microenvironment co-cultures were either left untreated (Co.), treated with curcumin alone (5µM), 5-FU alone (1, 5, and 10µM) or were pretreated for 4 h with curcumin (5µM) followed by treatment with 5-FU (0.1, 1, 2, 3µM). After 10 days of culture, total cell lysates of HCT116 high density cultures were prepared and analyzed by western blotting and quantitative densitometry for CSC marker ALDH1 (a), CD133 (b) and CD44 (c). Densitometric evaluation of protein expression as revealed by western blot analysis was performed in triplicate. Housekeeping protein β-actin served as a loading control in all experiments. Values were compared to the control and statistically significant values with *p*<0.05. Significant values are marked with (*).

Next, CSC markers (CD133, CD44 and ALDH1) expression was examined for tumor formation capacity and the chemosensitization effect of curcumin on CSC markers in HCT116 3D high density tumor microenvironment co-cultures. The co-cultures were either left untreated, treated with curcumin (5µM), 5-FU (1, 5, and 10µM) alone or were pretreated for 4 h with curcumin (5µM) followed by treatment with 5-FU (0.1, 1, 2, 3µM) for 10 days (Fig. 4). Additionally, in another set of experiments, HCT116 cells were incubated in high density mono-cultures, without fibroblasts as a basal control. Control high density mono-cultures of HCT116 showed basal expression of CSC markers (Fig. 4:a,b,c). In contrast to this, immunoblotting analysis of whole cell lysates showed marked up-regulation of CD133, CD44 and ALDH1 in HCT116 in control and in 5-FU-treated high density tumor microenvironment co-cultures in a concentration-dependent manner (Fig. 4:a,b,c). However, interestingly pre-treatment with curcumin followed by treatment with 5-FU significantly down-regulated CSC marker expression in a concentration-dependent manner on HCT116 cells in high density tumor co-cultures (Fig. 4:a,b,c). Densitometric analysis of typical western blot experiments show down regulation of CSC markers in HCT116 cells in cultures treated with either 5-FU, curcumin or/and 5-FU curcumin (Fig. 4:a,b,c). Taken together, these results indicate that the paracrine interaction between tumor and stromal cells is crucial in promoting CSCs and that there is a strong chemosensitizing effect of curcumin on colon CSC in high density tumor microenvironment co-cultures.

### Curcumin and/or 5-FU interfere in the synergistic crosstalk between CRC-/CSC-cells and fibroblasts in high-density tumor microenvironment co-cultures

**Figure 5:**
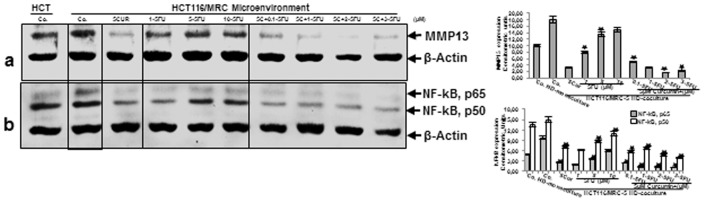
Effects of 5-FU, curcumin and the combinational treatment on proliferation-, metastatic gene products and NF-κB expression in the high density tumor microenvironment co-culture. HCT116 high density mono-cultures were either left untreated (HCT, Co.) or were co-cultured with MRC-5 in monolayer. Tumor microenvironment co-cultures were either left untreated (Co.), treated with curcumin alone (5µM), 5-FU alone (1, 5, and 10µM) or were pretreated for 4 h with curcumin (5µM) followed by treatment with 5-FU (0.1, 1, 2, 3µM). After 10 days of culture, total cell lysates of HCT116 high density cultures were prepared and analyzed by western blotting and quantitative densitometry for MMP-13 (a) and NF-κB (b). Densitometric evaluation of protein expression as revealed by western blot analysis was performed in triplicate. Housekeeping protein β-actin served as a loading control in all experiments. Values were compared to the control and statistically significant values with *p*<0.05. Significant values are marked with (*).

To examine the interaction between CRC-/CSC-cells and fibroblasts and to evaluate the effect of curcumin and/or 5-FU on this synergistic crosstalk, on tumor cell proliferation, invasion and tumor-promoting factors (MMPs, NF-κB) expression in more detail, we performed western blotting analysis of the high density tumor microenvironment co-cultures. The co-cultures were either left untreated or treated as described above (Fig.4). Untreated high density mono-cultures of HCT116 expressed MMP-13, however compared to HCT116 high density tumor microenvironment co-cultures, expression was markedly lower (Fig. 5:a). These results are in agreement with other studies that expression of MMPs is known to be up-regulated in the tumor microenvironment [17]–[19], [41], known to be regulated by transcription factor NF-κB [36], [42]. Treatment of HCT116 high density tumor microenvironment co-cultures with curcumin down-regulated the expression of MMP-13 (Fig. 5:a). In contrast, immunoblotting analysis of HCT116 treated with 5-FU showed marked dose dependent up-regulation of MMP-13 (Fig. 5:a). The pre-treatment of the HCT116 tumor microenvironment co-cultures with curcumin followed by treatment with 5-FU, although it contains only low concentrations of 5-FU compared to 5-FU treatment alone, proved to be most effective in down-regulation of the above mentioned protein in a concentration-dependent manner (Fig. 5:a). The mechanism involved in curcumin-mediated inhibition of 5-FU-induced proliferation and metastatic gene products in HCT116 cells, which are regulated by NF-κB was further examined. Moreover, it has been shown that NF-κB mediates tumor progression, and the tumor microenvironment is known to activate NF-κB expression [24], [43]. To evaluate whether curcumin inhibits the 5-FU-induced activation of NF-κB, HCT116 were probed for the phosphorylated form of the p65/p50 subunits. The results showed that significantly more activation of p65 subunit was observed in HCT116 high density tumor microenvironment co-cultures compared to control HCT116 high density mono-cultures and 5-FU-induced p65/p50 phosphorylation in a concentration-dependent manner (Fig. 5:b). Indeed, curcumin blocked 5-FU-induced phosphorylation of p65/p50 subunits in a concentration-dependent manner. Interestingly, co-treatment of the cultures with combinations of the two agents increased these effects more than each agent by itself (Fig. 5:b). Densitometric analysis of typical western blot experiments show down regulation of NF-κB and MMP-13 in HCT116 cells treated with either 5-FU, curcumin or/and 5-FU (Fig. 5:a,b). Taken together, these data suggest that fibroblasts promote tumor cells progression in the co-culture microenvironment, at least in part through NF-κB pathways and this could be blocked by curcumin, thereby sensitizing CSCs to 5-FU treatment.

### Inhibition of TGF-β3 and TGF-β3R expression in human colon cancer cells by 5-FU or curcumin in high density cultures

**Figure 6:**
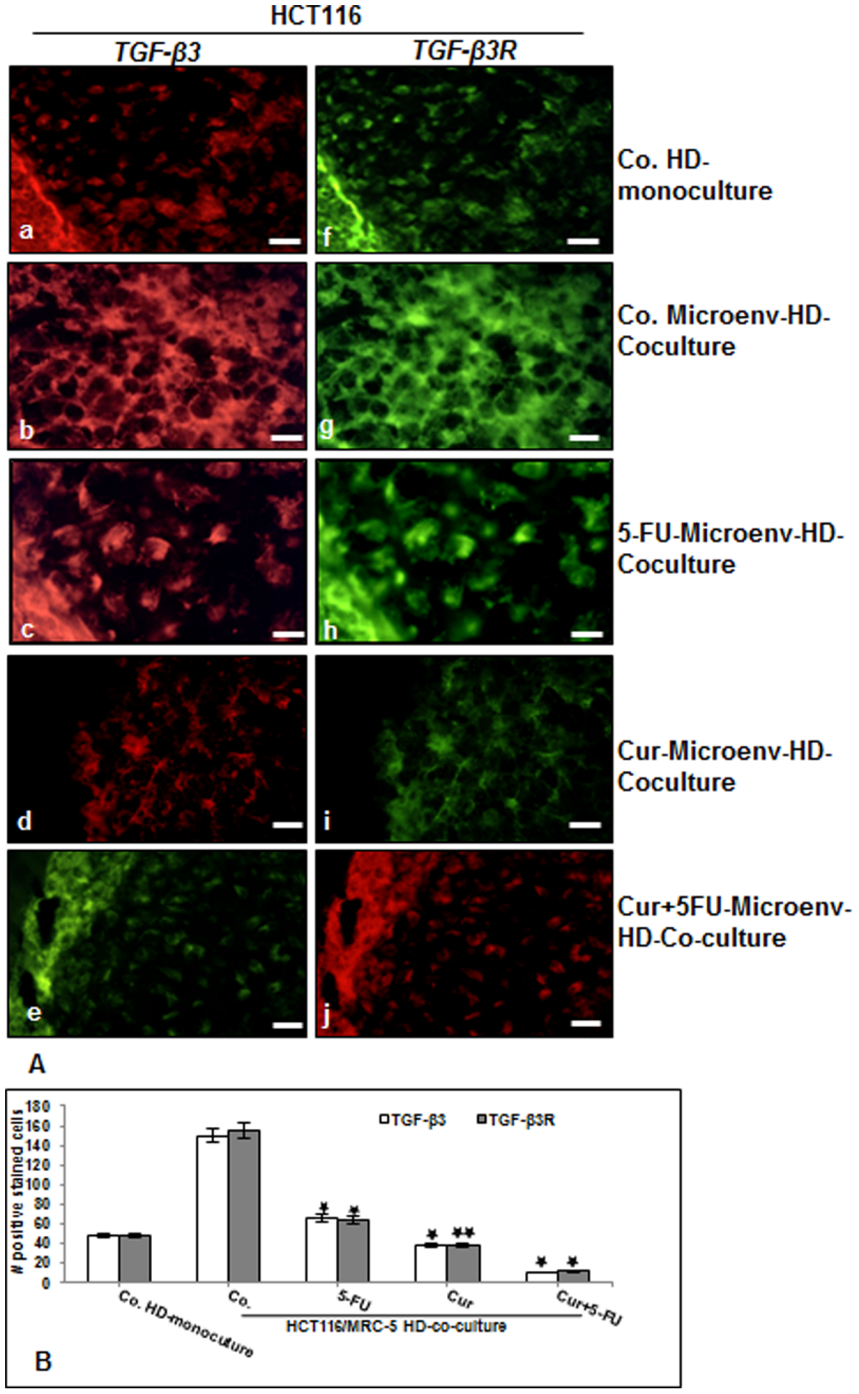
Curcumin or 5-FU suppresses TGF-β3 and TGF-βR expression in CRC cells in high density tumor microenvironment co-culture. A: High density mono-cultures of HCT116 cells were left untreated, high density tumor microenvironment co-cultures of HCT116/MRC-5 cells were either left untreated, or treated with 5-FU (5µM), or with curcumin (5µM) or pre-treated with curcumin (5µM) for 4 h, and then exposed to 5-FU (0.1µM) for 10 days. The cultures were subjected to immunofluorescence labeling with primary antibodies for TGF-β3 (a-e) and TGF-β3R (f-j) followed by incubation with rhodamine- or FITC-coupled secondary antibodies. Images shown are representative of three different experiments. Magnification 400×. bar 30 nm. B: To quantify the amount of TGF-β3 and TGF-βR-positive cells in high density cultures described above, 200 cells from 15 microscopic fields within the stained slides were counted. The results are provided as the mean values with S.D. from three independent experiments. Values were compared with the control and statistically significant values with *p<*0.05 were designated by an asterisk (*) and *p<*0.01 were designated by an asterisk (**).

To further characterize the potential roles of paracrine factors in the process of the synergistic crosstalk in cancer-stromal cells interaction, we next examined TGF-β expression in HCT116 to explore whether TGF-β is involved in enhancing tumor cell proliferation and tumor-promoting factors. The co-cultures were either left untreated or treated as described above (Fig. 4). The cultures were subjected to immunofluorescence labeling with primary antibodies for TGF-β3 and TGF-β3R followed by incubation with rhodamine- or FITC-coupled secondary antibodies. The expression of TGF-β3 and TGF-β3R was found diffusely distributed on the round HCT116 cells cultured in basal control high density mono-cultures (Fig. 6A: a,f). Interestingly, in contrast to this, expression of TGF-β3 and TGF-β3R in HCT116 cells in high density tumor microenvironment co-cultures was higher compared to that in control mono-culture (Fig. 6A: b,g), indicating the important role of the crosstalk in HCT116 and MRC-5 cells in co-cultures for tumor promotion. In HCT116 tumor microenvironment co-cultures treatment with 5-FU slightly up-regulated the expression of TGF-β3 and concomitantly TGF-β3R (Fig. 6A: c,h). In contrast, TGF-β3 and TGF-β3R expression in the HCT116 cell population significantly decreased upon treatment with curcumin or/and 5-FU treatment (Fig. 6A: d-j). These results suggest that the co-culture of tumor cells and fibroblast cells stimulates TGF-β expression/activation in fibroblasts and that in turn activates further the tumor cells. Statistical evaluation, as shown in Fig. 6B, confirmed the results in Fig. 6A.

### The synergistic crosstalk between CRC-cells and fibroblasts in tumor microenvironment co-cultures is mediated partially by TGF-β signaling

**Figure 7:**
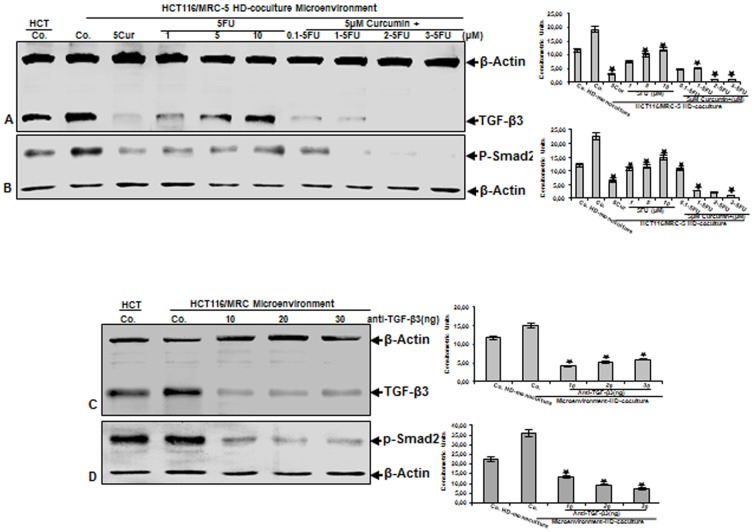
Curcumin, 5-FU and the combinational treatment suppress TGF-β-mediated synergistic crosstalk between CRC-cells and fibroblasts in tumor microenvironment co-cultures. A-B: HCT116 high density mono-cultures were either left untreated (HCT, Co.) or were co-cultured with MRC-5 in monolayer. Tumor microenvironment co-cultures were either left untreated (Co.), treated with curcumin alone (5µM), 5-FU alone (1, 5, and 10µM) or were pretreated for 4 h with curcumin (5µM) followed by treatment with 5-FU (0.1, 1, 2, 3µM). After 10 days of culture, total cell lysates of HCT116 high density cultures were prepared and immunoblotting performed for TGF-β3 (A) or p-Smad2 (B). C-D: HCT116 high density mono-cultures were either left untreated (HCT, Co.) or were co-cultured with MRC-5 in monolayer. Tumor microenvironment co-cultures were either left untreated (Co.) or treated with neutralizing pan-TGF-β antibody (10, 20, 30 ng/ml). After 10 days of culture, total cell lysates of HCT116 high density cultures were prepared and immunoblotting performed for TGF-β3 (C) or p-Smad2 (D). Densitometric evaluation of protein expression as revealed by western blot analysis was performed in triplicate. Housekeeping protein β-actin served as a loading control in all experiments. Values were compared to the control and statistically significant values with *p*<0.05. Significant values are marked with (*).

To further examine TGF-β signaling pathway in CRC cells in the tumor microenvironment, the co-cultures were either left untreated or treated as described above (Fig. 4). Immunoblotting of cell lysates of the HCT116 high density tumor microenvironment co-cultures indicated that more TGF-β3 was expressed compared with HCT116 from tumor mono-cultures (Fig. 7A), as seen as broad bands with apparent molecular weights ranging over 25 kDa, which are characteristic of TGF-β3 polypeptide. Treatment of HCT116 with curcumin by itself significantly down-regulated TGF-β3 protein expression (Fig. 7A). In contrast, HCT116 treatment with 5-FU dose-dependently up-regulated production of TGF-β3 protein and this was markedly down-regulated again through combinational curcumin/5-FU treatment (Fig. 7A). Next, we investigated whether TGF-β signaling pathway activation is related to the induction of Smad2 (Fig. 7B) in HCT116 cells treated as described above. Indeed, several studies showed that the most important signal transducers for the transmission of TGF-β intracellular signaling are the Smads with the ability to propagate signals from the activated receptor complex to the nucleus [44]–[46]. Western blot analysis showed that Smad2 phosphorylation was significantly higher in HCT116 tumor microenvironment co-culture compared with HCT116 high density mono-cultures (Fig. 7B), as seen as broad bands with apparent molecular weights ranging over 58 kDa, which are characteristic of p-Smad2 polypeptides. Following curcumin treatment, significantly less Smad2 phosphorylation was observed, whereas treatment with 5-FU dose-dependently increased Smad2 (Fig. 7B). Interestingly, with combination curcumin/5-FU treatment Smad2 phosphoylation was inhibited in a dose-dependent manner. Taken together, these results demonstrate a potential adaptation and resistance of CRC/CSC cells towards increasing dosages of 5-FU and clearly demonstrate the positive chemosensitization effect through curcumin to 5-FU by the combinational treatment. Further, these data demonstrate that canonical TGF-β signaling cascades are activated upon stimulation of CRC/CSC cells and stromal fibroblasts in the tumor microenvironment co-cultures. We next asked whether TGF-β signaling pathway is pivotal for the process of the synergistic crosstalk in cancer-stromal cells interaction. High density tumor microenvironment co-cultures were either left untreated, treated with a neutralizing pan-TGF-β antibody or control IgG as described in Materials and Methods for 10 days (Fig. 7C-D). Culturing of stromal cells with HCT116 cells in high density tumor microenvironment co-cultures resulted in an increase of TGF-β expression and Smad2 phosphorylation in HCT116 compared to high density tumor mono-cultures, and this response was considerably blocked by the neutralizing pan-TGF-β antibody (Fig. 7C-D), but not by control IgG (not shown). Taken together, these results indicate that tumor cells in the microenvironment co-cultures, can activate fibroblasts to synthetize active TGF-β, as part of a paracrine interaction in the microenvironment medium that in turn induces tumor cell activation, promoting progression and expanded metastatic patterns, increasing thereby the malignancy of the cancer cells.

### The intensive crosstalk between HCT116 and MRC-5 cells induces EMT in tumor microenvironment co-cultures

**Figure 8:**
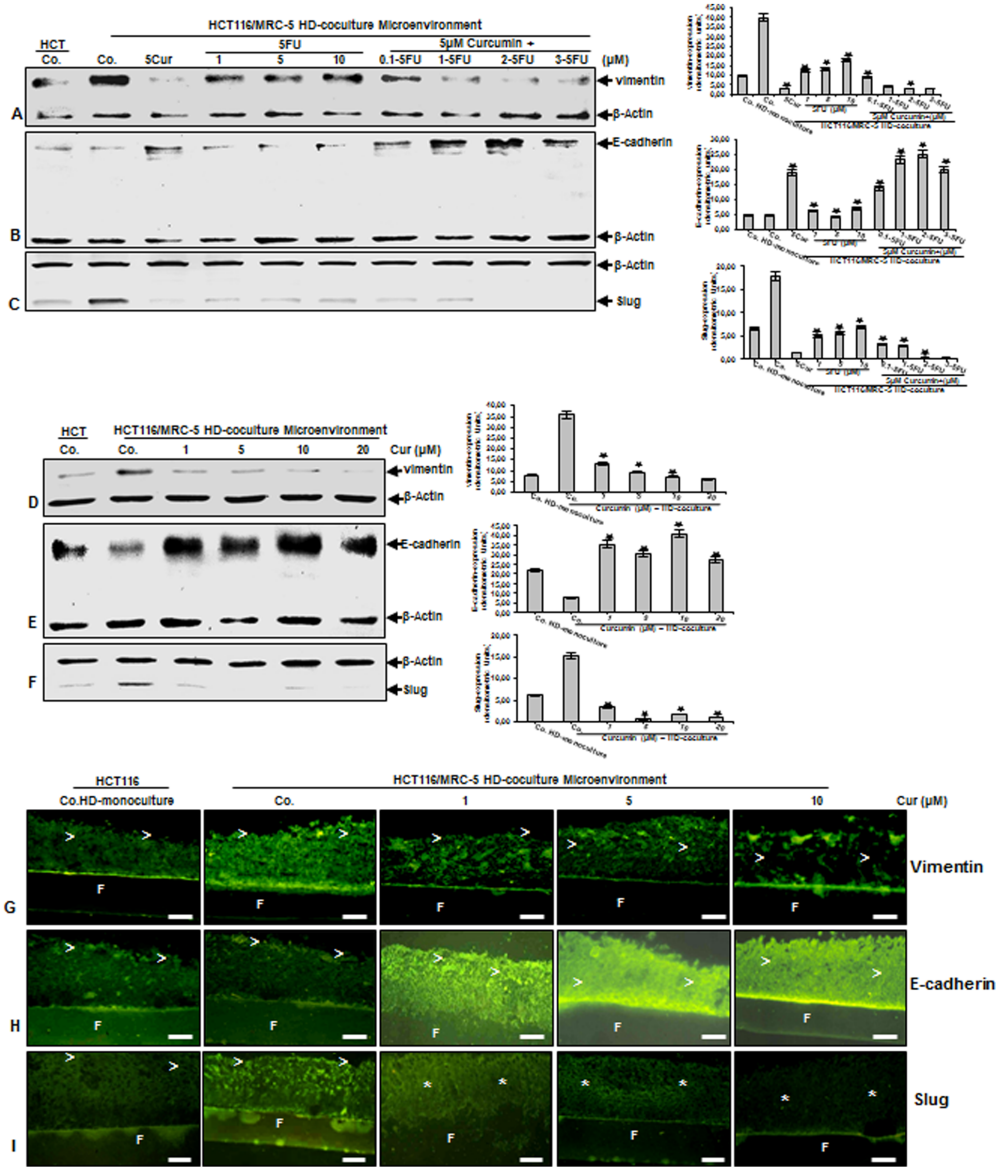
High density tumor microenvironment co-cultures induce EMT with loss of epithelial markers and increase of mesenchymal markers. A-C: HCT116 high density mono-cultures were either left untreated (HCT, Co.) or were co-cultured with MRC-5 in monolayer. Tumor microenvironment co-cultures were either left untreated (Co.), treated with curcumin alone (5µM), 5-FU alone (1, 5, and 10µM) or were pretreated for 4 h with curcumin (5µM) followed by treatment with 5-FU (0.1, 1, 2, 3µM). After 10 days of culture, total cell lysates of HCT116 high density cultures were prepared and immunoblotting performed for vimentin (A) or E-cadherin (B) or Slug (C). D-I: HCT116 high density mono-cultures were either left untreated (HCT, Co.) or were co-cultured with MRC-5 in monolayer. Tumor microenvironment co-cultures were either left untreated (Co.), or treated with curcumin (1, 5, 10, 20 µM). After 10 days of culture, total cell lysates of HCT116 HD-cultures were prepared for immunoblotting (D-F) or immunofluorescence performed on sections (G-I) for vimentin (D, G) or E-cadherin (E, H) or Slug (F, I). Densitometric evaluation of protein expression as revealed by western blot analysis was performed in triplicate. Housekeeping protein β-actin served as a loading control in all experiments. Values were compared to the control and statistically significant values with *p*<0.05. Significant values are marked with (*). F =  Filter. (>) =  HCT116 cells. Magnification G-I: 200×, bar = 30 nm.

**Figure 9:**
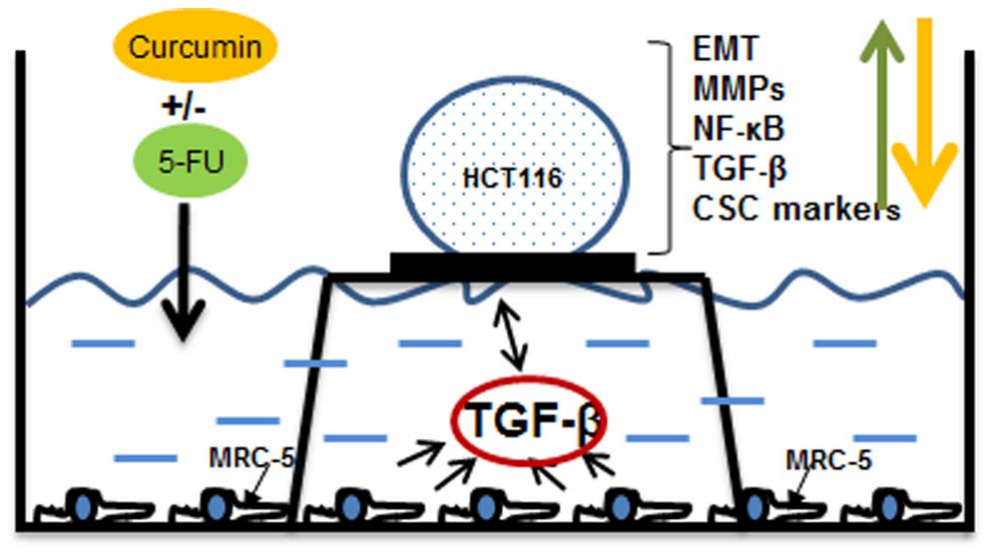
Schematic demonstrating the crosstalk between CRC-cells and fibroblasts in high density tumor microenvironment co-cultures. A 10µl drop of cell suspension containing around 1 million HCT116 cells is placed on a nitrocellulose filter on top of a steelnet bridge and the cells are nurtured by diffusion. MRC-5 cells are grown in monolayer on the bottom of the petri dish. This model mimics a three dimensional *in vivo* situation and allows the exchange between resident components and the cancer cells in the tumor microenvironment on the air medium interphase. Addition of therapeutic agents such as curcumin, 5-FU or neutralizing pan-TGF-β3 antibody can interact and influence cell signaling in both cell types influencing tumor cell and tumor stem cell proliferation, malignity and EMT.

In order to get further insights into the functional roles of paracrine factors in the process of the synergistic crosstalk in relation to EMT [47], [48], we have analyzed the expression of EMT-associated signaling molecules, such as vimentin, E-cadherin, Slug. The co-cultures were either left untreated or treated as described above (Fig. 4). Untreated high density mono-cultures of HCT116 expressed vimentin at markedly lower levels and higher E-cadherin expression compared to HCT116 high density tumor microenvironment co-cultures (Fig. 8A-B). Treatment of HCT116 tumor microenvironment co-cultures with curcumin down-regulated the expression of vimentin, whereas it increased E-cadherin levels (Fig. 8A-B). In contrast, immunoblotting analysis of HCT116 tumor microenvironment co-cultures treated with 5-FU showed marked dose dependent up-regulation of vimentin, but down-regulation of E-cadherin (Fig. 8A-B). Furthermore, EMT is associated with the development of increased resistance to chemotherapeutic agents [30]. To address this issue, we examined whether curcumin can modulate the expression of 5-FU-induced vimentin or E-cadherin. The pretreatment of the co-cultures with curcumin followed by treatment with 5-FU, proved to be most effective in down-regulation of vimentin or up-regulation of E-cadherin in a concentration-dependent manner in HCT116 (Fig. 8A-B). The transcription factor, Slug is associated in transcriptional suppression of E-cadherin expression and activation of vimentin in the molecular EMT program [34]. Interestingly, we observed a significant increase of Slug expression in HCT116 tumor microenvironment co-cultures compared with HCT116 tumor mono-cultures and this response was considerably blocked by curcumin (Fig. 8C). Furthermore, treatment of HCT116 tumor microenvironment co-cultures with 5-FU induced Slug expression in a concentration-dependent manner, whereas curcumin blocked 5-FU-induced Slug expression (Fig. 8C). Next, we asked whether EMT signaling pathway is one of the essential important processes of the synergistic crosstalk in cancer-stromal cells interaction and can be specifically influenced by curcumin. High density tumor microenvironment co-cultures were either left untreated or treated with curcumin (1, 5, 10, 20 µM) for 10 days (Fig. 8D-F). Culturing of stromal cells with HCT116 cells in the microenvironment co-culture resulted in an increase of vimentin and decrease of E-cadherin expression in HCT116 cells compared to tumor mono-cultures and the expression of vimentin was considerably inhibited and concomitantly the expression of E-cadherin increased by curcumin in a concentration-dependent manner (Fig. 8D-E). Consistent with the vimentin- and E-cadherin levels in high density tumor microenvironment co-cultures, we noted that Slug expression was increased in high density tumor microenvironment co-culture, whereas Slug expression decreased predominantly by curcumin in HCT116 in a concentration-dependent manner (Fig. 8F). Moreover, immunofluorescence microscopy confirmed the qualitative changes in the different specific EMT-markers observed in HCT116 cells by western blot analysis of whole-cell extracts (Fig. 8G-I). Taken together, these results suggest that the microenvironment co-cultures (Fig. 9) can activate EMT, as one important functional part of the synergistic crosstalk in cancer-stromal cells interaction, promoting progression and expanded metastatic patterns in the microenvironment and inhibition of this interaction by curcumin induces biochemical and functional changes towards MET, thereby sensitizing CSCs to 5-FU treatment.

## Discussion

The aim of this study was to investigate the effect of curcumin and/or 5-FU on CRC cell proliferation, tumor-promoting factors, EMT and cancer stem cells in an *in vitro* two- and three-dimensional tumor microenvironment co-culture model that mimics *in vivo* functions of CRCs/fibroblast tumor microenvironment interaction.

A large body of literature indicates that, crosstalk in the tumor microenvironment between cancer and stromal cells can promote survival, proliferation, malignant behavior of tumor cells and their ability to develop drug resistance [49]–[51]. In this study, we firstly evaluated the crosstalk between HCT116 and MRC-5 cells in monolayer co-cultures. A dynamic interaction occurred between two cells, and CRC cells proliferated around the fibroblasts, searching and establishing close cell-cell contact. Furthermore, we found that this direct interaction in the co-cultures stimulated the cells to enhance the promotion of metastatic adhesion molecules (β1-integrin, ICAM-1), proliferative proteins (cyclin D1, Ki-67), TGF-β3, p-Smad2 and vimentin, suggesting a dynamic interaction between the two cell types in the tumor microenvironment. Our findings correlate with results from other groups that have previously demonstrated that tumor cell and stromal cell crosstalk plays an important role in the initiation and promotion of cancer by secreting and exchanging cytokines and growth factors in the tumor microenvironment [39], [52], [53].

We next examined the crosstalk between CRCs and fibroblasts by indirect high density co-culture experiments that mimic the interaction between these cells in the tumor microenvironment *in vivo* (Fig. 9). In these co-culture experiments CRCs formed well defined high density tumor spheres and treatment with 5-FU and/or curcumin resulted in high cellular curcumin uptake and colonosphere disintegration. These results are in accordance with results from previous studies from our laboratory [12], [37], and others [11], [13] demonstrating the anti-tumor activity of curcumin and the chemosensitizing effect of curcumin in combination with 5-FU on CRC cells. Moreover it has been reported that curcumin exhibits synergistic activity with 5-FU also in other tumor cells [54], [55].

Next, we found with immunofluorescence evaluation that expression of stem cell marker (CD133) markedly increased in HCT116 in high density microenvironment co-cultures compared to HCT116 high density mono-cultures. This underlines the above findings, that the tumor microenvironment is a dominant factor that stimulates and supports formation of tumor stem cells. Simultaneously we could show that treatment with 5-FU and/or curcumin inhibited and even diminished stem cell marker expression in HCT116. As CSCs are believed to be mainly responsible for treatment resistance, treatment failure and tumor recurrence [6], [56], [57], this demonstrates that for new treatment approaches attention should be turned on targeting specifically the interaction of the microenvironment and the colon CSCs.

The promising results from the immunofluorescence led us to investigate more specifically signaling proteins, which influence the interactions in tumor microenvironment co-cultures, according to the regulation of tumor promoting inflammation factors and CSC marker in CRCs. In accordance with the consideration that the cells of the tumor stroma are a key determinant in tumor promotion [58], we found that tumor microenvironment co-cultures produced strikingly higher amounts of invasion related proteinase (MMP-13), had markedly higher activation levels of NF-κB compared to CRC high density mono-cultures and significant up-regulation of CSC markers expression. These results underline the importance of paracrine interaction between tumor and stromal cells as a crucial determinant of tumor progression, invasion and metastasis. Indeed, it has been reported that in the tumor microenvironment between the tumor and stroma paracrine and autocrine communications play an important role, increasing the invasion and metastasis potential in tumor cells [58]–[60]. Interestingly, substantial production of tumor promoting factor (MMP-13) and marked levels of NF-κB were further pronounced in the presence of 5-FU. Moreover, many different tumor cells activate constitutive inducible anti-apoptotic NF-κB for their survival [43], [61]. This result seems to be paradox at first, as 5-FU would be expected to impede tumor promotion. However, taken into consideration that 5-FU also stimulated enrichment of CSC marker positive cells in high density tumor microenvironment co-cultures, these results might be regarded as a defense reaction/response of the tumor cell culture and the tumor microenvironment. In fact, it has been reported that in contrast to the differentiated population of tumor cells, cancer stem cells are characterized by increased resistance to cytotoxic chemotherapeutic agents [62], enhanced ability to form colonospheres [6], [13], [63] and induce remission, providing basis for why cancer cells cannot be completely destroyed by conventional chemotherapeutic agents [57], [64]. Indeed, we could further show that combinational treatment of curcumin and 5-FU dramatically suppressed tumor promoting factors, activation of NF-κB signaling pathway and enrichment of CSCs in high density tumor microenvironment co-cultures. These results demonstrate once more the potent chemosensitizing effect of curcumin on CRC, and to our knowledge show for the first time the modulating effect of curcumin on the crosstalk between the CRC/CSCs and the fibroblasts in the tumor microenvironment, creating an adequate climate for more effective chemotherapeutic action of 5-FU to specifically target chemoresistant colorectal stem cells.

It seems that for prevention and treatment of tumor development, it is important to find factors supporting cancer microenvironment. We found in the present study either in monolayer or in high density tumor co-cultures that the intensive crosstalk in the tumor microenvironment co-cultures increased TGF-β3 and p-Smad2 levels in HCT116 cells compared to mono-cultures, indicating active TGF-β signaling in these cells in the tumor microenvironment. Moreover, expression of TGF-β and phosphorylation of Smad2 was significantly decreased by treatment with curcumin. To see that TGF-β secretion as one possible paracrine factor plays an important role in the microenvironment co-culture, the expression of TGF-β and phosphorylation of nuclear p-Smad2 could be significantly inhibited by neutralizing pan-TGF-β antibody, indicating a strong TGF-β dependency. Interestingly, it has been reported that TGF-β activates NF-κB and NF-κB-dependent inflammatory proteins in tumor cells [45]. These results are in agreement with other studies showing that TGF-β is the most prominent of the paracrine factors within the tumor microenvironment [65], [66].

Several lines of evidence have shown that tumor-associated fibroblasts, inflammatory cells, cytokines and EMT in tumor biology are potential factors that enable cancer cell invasion, metastasis and malignancy and this process is reversible [47], [67]. Tumor cells have a diversity of phenotypes, malignancy and they lose their epithelial properties during EMT development [30]. It has been reported that EMT is a triggering factor for tumor stem cell formation [68]. Furthermore, as observed, EMT is identified/characterized by the loss of epithelial properties, including down regulation of cell adhesion molecules like E-cadherin and at the same time promoting of N-cadherin, vimentin, fibronectin, zinc-finger proteins (SNAIL, Slug, ZEB) and matrix metalloproteinases (MMPs) expression, conducting the tumor cells to an higher cell mobility and malignity [48]. Moreover, It has been reported that the EMT-inducing factors, such as TGF-β, PI3K/AKT- and Wnt-signaling [69], [70] produced by the surrounding tumor microenvironment stromal cells [47] can influence the behavior and invasive phenotype in epithelial malignancies initiating of tumor cells. Our results in this study show that tumor microenvironment co-culture increased EMT markers, such as vimentin, slug and decreased E-cadherin in HCT116 cells compared to mono-cultures, indicating the high density tumor microenvironment co-cultures induces EMT. Expression of vimentin and slug was significantly decreased and E-cadherin was significantly increased by treatment with curcumin. Consistent with these results, it has been reported that MMPs or cytokines, which are secreted by TGF-β-stimulated stromal cells, initiate GP130/STAT3 and NF-κB signaling in cancer cells at early time points of the metastatic process and this crosstalk allows a tumor promoting microenvironment surviving metastatic tumor cells [29]. Indeed, both signaling pathways, NF-κB and TGF-β3, can also be activated by direct interactions between cancer cells and platelets and were found to promote EMT and metastasis [71]. Several studies have shown that in cancer an increase of mesenchymal characteristics (N-cadherin and vimentin) and loss of epithelial characteristics (E-cadherin) via EMT is proportional with cancer progression, motility, invasiveness, drug resistance and metastasis [72]–[75]. Thus, targeting EMT and inhibition of cancer stem cells may be a promising strategy to suppress metastasis and improve survival of cancer patients. Other different factors secreted by fibroblasts/tumor cells might also influence TGF-β effects on tumor cells differentiation in microenvironment co-culture, as has been shown for interleukin-1 [76], [77].

In conclusion, interaction between tumor cells and fibroblasts occur either direct or paracrine and thereby create a complex cellular tumor microenvironment (Fig. 9) regulating CSC promotion, their progeny and finally invasion and metastasis activity. The simultaneous targeting of pathways that regulate interaction of cell types resident in the tumor microenvironment and the colorectal CSC might eventually improve the efficacy of therapeutic outcome. The tumor microenvironment induces in the cells invasive properties through up-regulation of NF-κB, TGF-β, EMT, creating a tumor microenvironment feedback loop (Fig. 9). In this study, we could emphasize that the natural NF-κB inhibitor curcumin is a promising modulator of the synergistic crosstalk in the tumor microenvironment and curcumin-based anti-EMT and tumor progression may be a promising therapeutic strategy to prevent resistance to chemotherapeutic agents, sensitizing CSCs to 5-FU and impede metastasis formation.
